# Detection of intra-brain cytoplasmic 1 (BC1) long noncoding RNA using graphene oxide-fluorescence beacon detector

**DOI:** 10.1038/srep22552

**Published:** 2016-03-21

**Authors:** Mee Young Kim, Do Won Hwang, Fangyuan Li, Yoori Choi, Jung Woo Byun, Dongho Kim, Jee-Eun Kim, Kookheon Char, Dong Soo Lee

**Affiliations:** 1 Department of Nuclear Medicine, Seoul National University College of Medicine, Seoul, Korea; 2 Department of Molecular Medicine and Biopharmaceutical Sciences, Graduate School of Convergence Science and Technology, Seoul National University, Seoul, Korea; 3 The National Creative Research Initiative Center for Intelligent Hybrids, School of Chemical & Biological Engineering, The WCU Program of Chemical Convergence for Energy & Environment, Seoul National University, Seoul, Korea; 4 Genolution Inc., Asan Institute for Life Sciences, Korea

## Abstract

Detection of cellular expression of long noncoding RNAs (lncRNAs) was elusive due to the ambiguity of exposure of their reactive sequences associated with their secondary/tertiary structures and dynamic binding of proteins around lncRNAs. Herein, we developed graphene-based detection techniques exploiting the quenching capability of graphene oxide (GO) flakes for fluorescent dye (FAM)-labeled single-stranded siRNAs and consequent un-quenching by their detachment from GO by matching lncRNAs. A brain cytoplasmic 1 (BC1) lncRNA expression was significantly decreased by a siRNA, siBC1–1. GO quenched the FAM-labeled siBC1–1 peptide nucleic acid (PNA) probe, and this quenching was recovered by BC1. While FAM-siBC1–1-PNA-GO complex transfected spontaneously mouse or human neural stem cells, fluorescence was recovered only in mouse cells having high BC1 expression. Fluorescent dye-labeled single-stranded RNA-GO probe could detect the reactive exposed nucleic acid sequence of a cytoplasmic lncRNA expressing in the cytoplasm, which strategy can be used as a detection method of lncRNA expression.

Among noncoding RNAs, microRNA expression was easily traced using reporter transgenes and their mature form’s action was traced by ingeniously-designed reporter transgenes using appropriate targets on their 3′ UTR^1^,^2^. This achievement was enabled by the knowledge about primary microRNA production and its processing and action. However, long noncoding RNAs (lncRNAs) are difficult to trace for its action within the cells because of the complicated structures and their RNA binding proteins^3^,^4^.

In this study, brain cytoplasmic 1 (BC1) lncRNA was chosen as a candidate for lncRNA sensing, considering the advantages of its small size (154 nt), and cytoplasmic localization to which probes can get access easily. BC1 lncRNA is a brain-specific transcript whose expression is primarily localized to dendrites in rodents^5^. This lncRNA is particularly and rapidly transported to dendrites^6^ and inhibits translation initiation by interacting with eIF4A and poly(A)-binding protein (PABP)^7^. Loss of BC1 causes neuronal hyperexcitability^8^ and upregulates the synaptic proteins such as PSD-95 which is associated with susceptibility of epileptic seizures^9^. Interestingly, it was reported that the expression of BC200 (primate counterpart of BC1) was gradually declined between age 49 and 86, and BC200 level in the brain of Alzheimer’s disease patient was significantly higher than normal brain^10^.

The graphene oxide (GO) is known to be bound with dye-labeled single-stranded DNA (ssDNA) probe via π-stacking interaction and makes the fluorescent dye-labeled probe quenched^11^. Interestingly, fluorescence could be recovered after dye-labeled probe releases from GO by interaction with intracellular complementary target DNA^12^. Using these characteristics, GO was used as sensors to detect DNA^13^, microRNA^14^, metal ions^15^,^16^, and active enzyme^17^,^18^. More recently, microRNA-sensing techniques using a peptide nucleic acid (PNA) and nanographene oxide showed capability of GO as biosensor in living cells *in vitro*^19^.

Here, we developed the sensing techniques for the expression of BC1 lncRNA via siRNA-based probe selection. Specific probe to detect BC1 lncRNA was selected via siRNA-based knockdown test for BC1 lncRNA. GO that has relatively homogenous size was chosen as reliable carrier in GO-based lncRNA sensor (Fig. 1). We could delineate the efficacy of siPNA probe-GO system to sense intracytoplasmic BC1 lncRNA expression by signal-on way in live neural stem cells *in vitro*.

## Results

### Design of siRNA-based probe for BC1 lncRNA detection

**Figure 2:**
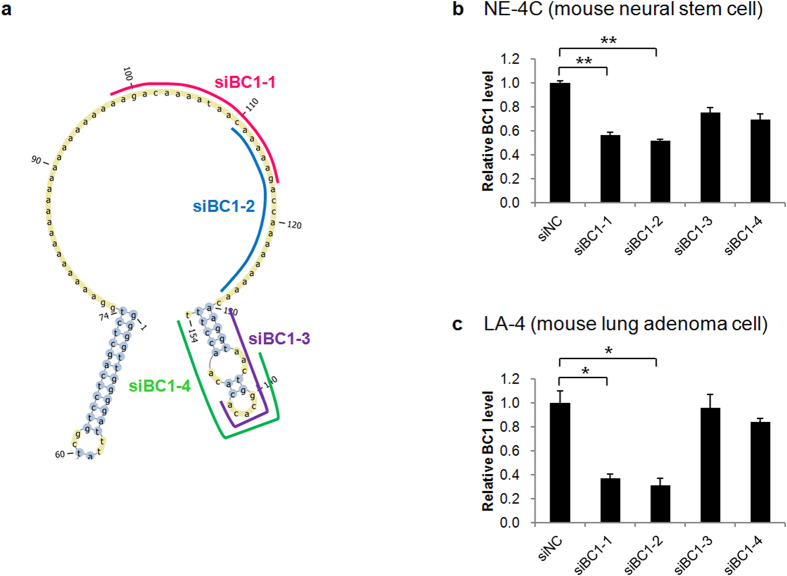
Design of BC1 lncRNA specific probes from siRNA-based approach. (**a**) Full length of BC1 lncRNA includes individual positions of four potential siRNA binding sites. (**b,c**) Real-time PCR analysis for BC1 expression was performed on cell groups treated with four independent BC1 siRNAs (siBC1–1, siBC1–2, siBC1–3 and siBC1–4) or negative control (siNC). BC1 positive NE-4C (**b**) and LA-4 (**c**) cells were used to find the optimal BC1 detection probe by confirming the knockdown of BC1 for the each designed siRNA. Data are represented as mean ± SD (n = 3). *P < 0.005 and **P < 0.001 (two-tailed Student’s *t* test).

The presence or absence of lncRNAs such as BC1 was easily examined using fractionated organelle qRT-PCR but the active functioning of the lncRNA in the intracellular milieu can only be observed by molecular imaging using fluorescent proteins, sometimes transduced and persistently expressing or in other times transiently transfected to the cells. The four siRNAs for BC1 lncRNA were designed to correspond to the nucleotides 98–116 (siBC1–1), 110–128 (siBC1–2), 127–144 (siBC1–3) and 136–154 (siBC1–4) (Fig. 2a). The siBC1–1 and siBC1–2 recognized the loop region of BC1 lncRNA, whereas siBC1–3 and siBC1–4 targeted the spanned stem region. These four siRNAs were transfected into the mouse neural stem cells NE-4C or the mouse lung adenoma LA-4 cells. The BC1 lncRNA expression decreased by siBC1–1 or siBC1–2 in comparison to the siNC in NE-4C cells (Fig. 2b, n = 3). In LA-4 cells, the BC1 lncRNA expression also decreased by siBC1–1 and siBC1–2 (Fig. 2c, n = 3). The siRNA-mediated reduction of BC1 lncRNA transcripts revealed siBC1–1 as the best candidate. We have selected another siBC1–3 to assess the difference of the probe by the efficiency of the siRNA. Each siRNA probe was modified as a PNA form to enhance the stability and sequence of PNA probe for BC1 lncRNA is in table supplement. As controls, we used the scrambled form of FAM-PNA-BC1-1 (FAM-PNA-scr1). Size was determined using a Nanoparticle Tracking Analysis after FAM-PNA-BC1–1 probe was loaded with GO sheet. The overall size distribution and maximum size peak between GO sheet and FAM-PNA-BC1–1-GO were not changed (Supplementary Figure S1).

### Specificity of FAM-PNA-GO complex

**Figure 3:**
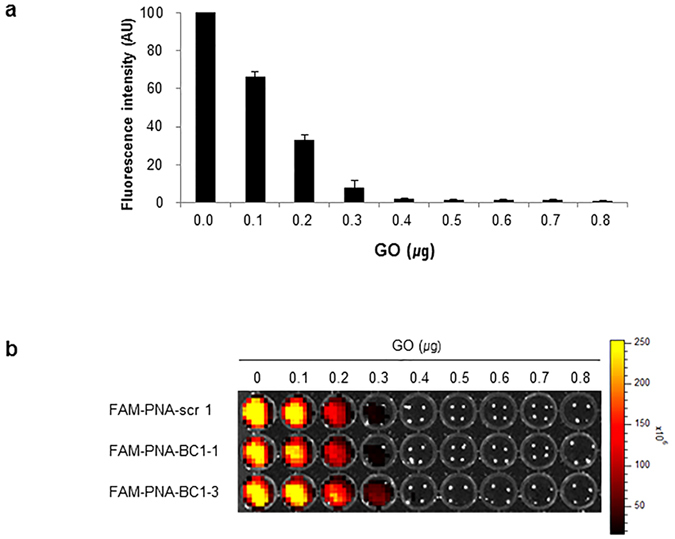
Quenched fluorescence intensity of FAM-PNA probes as different concentration of GO in tube. (**a**) FAM-conjugated PNA probes were reacted with or without GO in 50 mM Tris buffer (pH 7.5). Fluorescence signals of FAM-PNA probes were gradually quenched after incubation with different dose of GO within 10 minutes, measured by fluorometer. Data are represented as mean ± SD (n = 3). (**b**) The fluorescent plate images were taken in 96 well plates by using fluorometer device.

**Figure 4:**
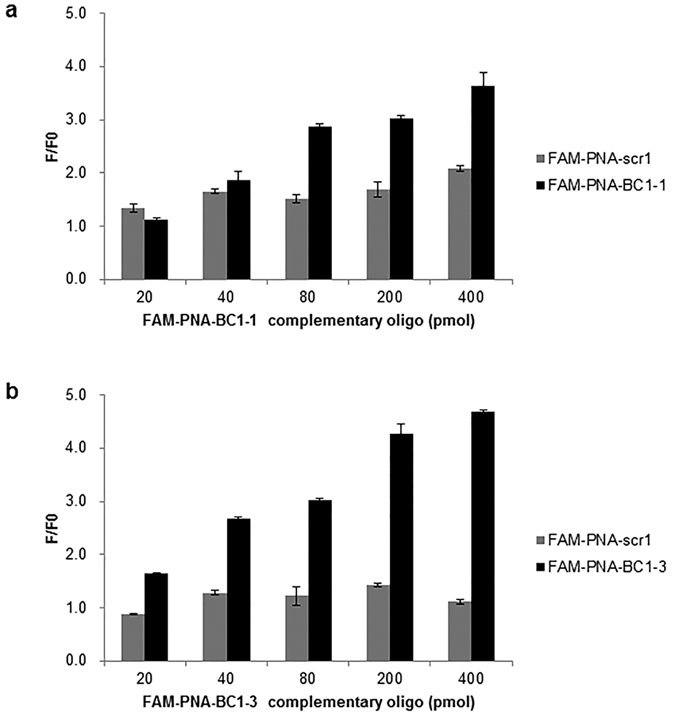
Specificity test of FAM-PNA-GO after treatment of complementary oligomer. (**a,b**) The fluorescence signals of FAM-PNA probe (40 pmol) were quenched by the addition of GO (0.4 μg) and then FAM-PNA-GO complexes were mixed with the complementary BC1–1 oligomer (**a**) and BC1–3 oligomer (**b**) at different concentrations (F, fluorescence intensity in the presence of oligomer; F0, basal fluorescence intensity in the absence of oligomer). The fluorescent signals were recovered as its matched complementary oligomer concentration was increased. Fluorescence plate images also showed the gradual increase in fluorescence signal as complementary oligomer was increased.

To find the optimal concentration of GO to maximize the fluorescence quenching efficacy, varying concentration of GO was mixed with FAM-PNA probes (40 pmoles), showing that 0.4 μg of GO completely quenched the fluorescence (Fig. 3a,b). Upon addition of increasing amount of complementary oligomer to the FAM-PNA-BC1-GO mixture, fluorescence was recovered only when FAM-PNA-BC1–1-GO complex was mixed with the complementary oligomer of BC1–1 (Fig. 4a). This was also the case with FAM-PNA-BC1–3-GO complex with the complementary oligomer of FAM-PNA-BC1–3 (Fig. 4b).

### Recognition of BC1 lncRNA by the FAM-PNA-BC1-GO complex in the cells

**Figure 5:**
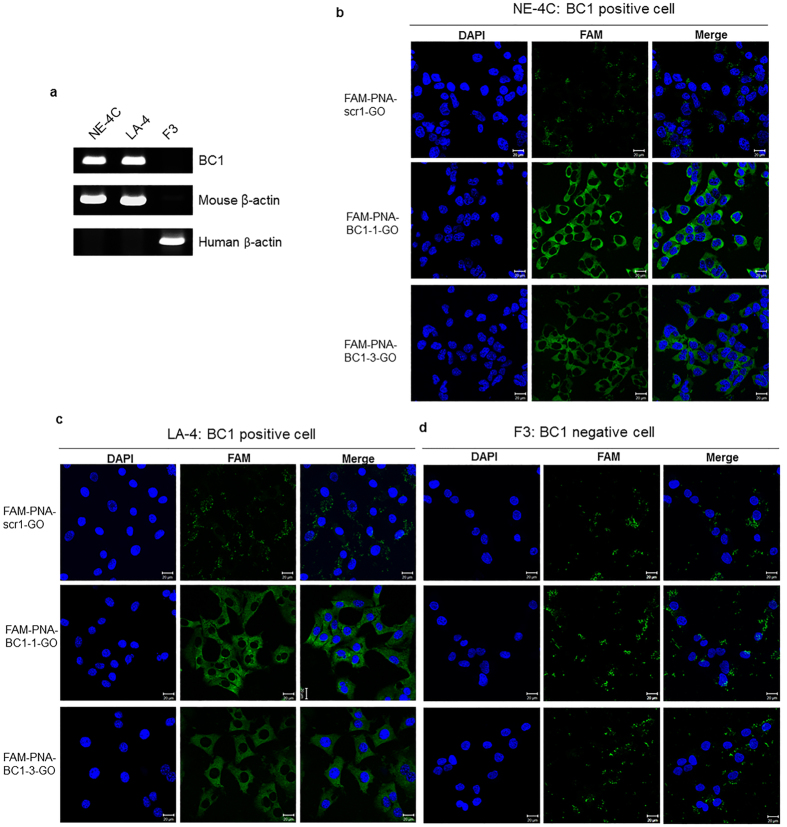
Detection of BC1 lncRNA by FAM-PNA-BC1-GO in three different cell lines. (**a**) Total RNA from individual cells was isolated and RNA expression was analyzed by RT-PCR. Mouse and human β-actin are used as a control. (**b–d**) Fluorescent images of the cells were taken at 14 h after treatment of FAM-PNA-GO (100 pmol) at 37 °C. Confocal microscope results showed that fluorescence signals were significantly high in the cytoplasmic area of NE-4C (**b**) and LA-4 (**c**) after treatment of FAM-PNA-BC1–1-GO and FAM-PNA-BC1–3-GO. No fluorescence signals were observed in FAM-PNA-scr1-treated group. Also, FAM-PNA-BC1-GO treatment showed few fluorescence signals were seen in BC1 negative F3 cell line (**d**). Scale bar: 20 μm. (Green, FAM signals from PNA probe; blue, DAPI signal from nuclei).

The RT-PCR showed that mouse NE-4C neural stem cells expressed highly the BC1 lncRNAs and that human F3 neural stem cell did not, but mouse LA-4 lung adenoma cells did (Fig. 5a). Thus, NE-4C and LA-4 cells were chosen as BC1 lncRNA positive cell lines, whereas F3 cell as BC1 lncRNA negative cell line. WST-8 assay to evaluate cytotoxicity of GO yielded that the viability of NE-4C and LA-4 cells decreased gradually as GO dose increased. GO did not exhibit cytotoxicity at the concentration (4 μg/mL) used in the present study (Supplementary Figure S2). In opti-MEM media as well as in the buffer, FAM-PNA were found to be completely quenched by adding GO (Supplementary Figure S3).

The intense fluorescence was found in the cytoplasm of the NE-4C cells after the incubation of 14 h treated with FAM-PNA-BC1–1-GO. No fluorescence was observed in the FAM-PNA-scr1-GO treated cells (Fig. 5b). The fluorescence of FAM-PNA-BC1–3-GO was also found in the cytoplasm but in very low intensity compared to FAM-PNA-BC1–1-GO, but slightly higher than that of FAM-PNA-scr1-GO treated group. Another BC1 lncRNA positive LA-4 cells also showed the similar cytoplasmic fluorescence with the treatment of FAM-PNA-BC1–1-GO and FAM-PNA-BC1-3-GO but not with FAM-PNA-scr1-GO (Fig. 5c). In contrast, in the BC1 lncRNA negative F3 cells, neither FAM-PNA-BC1–1-GO, FAM-PNA-BC1–3-GO nor FAM-PNA-scr1-GO showed any fluorescence signals (Fig. 5d). As controls, when the cells were incubated with FAM-PNA-BC1–1 alone, FAM-PNA-BC1–3 alone, FAM -PNA-scr1 alone or GO alone, no fluorescence was observed (Supplementary Figure S4a,b,c), which indicated that the selected FAM-PNA-BC1–1-GO probe specifically bound to BC1 lncRNA in BC1 lncRNA positive cells.

## Discussion

Amid the rapid emerging for significance of lncRNAs as a biomarker or therapeutic target, sensing technique for lncRNAs is urgently needed. In this study, as a proof-of concept study, we introduced the PNA-formed probe of lncRNAs to enhance physiological stability for lncRNA detection in cellular level. Until recently, molecular beacon (MB) system is constructed based on the fluorescence recovery of quenched fluorescent molecule in the presence of sequence-specific target molecules^20^. Despite MB’s substantially high sensitivity and specificity, nuclease degradation and temperature-sensitive non-specific fluorescence recovery limits its efficacy. MBs made by 2-OMe-modified RNA, PNA, or locked nucleic acid (LNA) can protect these targeting nucleic acids against nuclease cleavage. In this study, we adopted the PNA probe for lncRNAs to enhance the stability in the cells. Unlike black-hole quencher incompletely quenching the fluorescence of MB to detect mature microRNA during neuronal differentiation^21^, GO was reported to quench almost completely the fluorescence of MB making the cellular background minimized. In addition, GO made MB resistant to nuclease and cytotoxicity of MB-GO become lower than MB themselves^22^.

PNA-GO in one study successfully detected multiple microRNAs in living cells and even could monitor target microRNAs quantitatively^19^. MicroRNA-complementary sense sequence was used as a microRNA probe to detect the endogenous presence of mature microRNAs. In our study, the lncRNA-complementary sense sequence was not used as a probe, and instead, considering the fact that lncRNA is composed of three dimensional structures^3^,^4^, we needed to predict the open nucleotide frame and produce the siRNA-based probe against BC1 lncRNA.

PNA-BC1–1 was the one showing the best empirical performance to reduce the amount of intracytoplasmic BC1 lncRNA, PNA of which was neutrally charged enabling stable interaction with the surface of GO with minimal non-specific binding. GO from commercial sources whose size were variable with several orders of magnitude did not work, especially the fluorescence was not recovered by BC1 sense oligomers by any of FAM-PNA-BC1-GOs or control FAM-PNA-scr. When we used relatively uniform-sized GO collected via centrifugation method, fluorescence was recovered by the addition of matching sense oligomers in a dose dependent manner, compared to fluorescence signals in FAM-PNA-scr (Fig. 4a,b). In the cell study, this selected FAM-PNA-BC1-1-GO probe could be specifically switched on in the cytoplasmic area of BC1 positive neural stem cells at 14 h after cell transfer of BC1 lncRNA sensor, indicating that FAM-PNA-BC1–1 can now be used as sensitive probe for BC1 lncRNA presence in live cells *in vitro*.

GO are now known to be internalized via almost all mechanism including caveolae, clathrin, macropinocytosis, or translocation^23^,^24^,^25^. We exploited the characteristics of GO internalizing across cellular plasma membrane and it worked in three different cells such as NE-4C, LA-4 and F3 cells. However, GO seems not to penetrate the nuclear membrane or pass through the nuclear pore complex, showing that GO was mostly observed to be localized in the cytoplasm^26^,^27^,^28^. As many lncRNAs participate in transcriptional regulation inside the nucleus, we need to develop the methods to deliver the FAM-PNA-GO complex to the nucleus^29^,^30^,^31^. Nuclear brain specific lncRNA such Gomafu and NEAT1 will then be studied for their action.

Detecting method of the functionally active endogenous lncRNA with its open frame which can be monitored by FAM-PNA-GO complex provides useful tool for further understanding how lncRNAs are working in intracellular milieu. This technology can now be used for the investigation of the roles of lncRNA using cellular models *in vivo* to understand neurodevelopmental and neurological diseases^32^,^33^. This method can be applied for the evaluation of the new reagents affecting the action of target lncRNAs by just examining their production (presence or absence) and RNA-binding protein-medicated open-and-closure of the open active frames (dynamic action) of the sequences of lncRNAs. This technique will be also helpful to study the sponge effect^34^,^35^,^36^ of lncRNAs against microRNAs to examine antagonistic/synergistic action^37^ between microRNA and lncRNAs during various cellular processes.

## Methods

### Cell culture and transfection

Mouse NE-4C neural stem cells were cultured in Eagle’s Minimum Essential Medium (EMEM) supplemented with 10% fetal bovine serum (FBS; Gibco, Grand Island, NY). Mouse LA-4 lung adenoma cells were cultured in RPMI 1640 with 10% FBS. Human F3 (HB1.F3) neural stem cells were in DMEM/F-12 (1:1) with 10% FBS. Cells were maintained at 37 °C in a humidified atmosphere containing 5% CO_2_. Transfection of cells was performed using lipofectAMINE2000 transfection reagent (Invitrogen, Carlsbad, CA).

### Synthesis of siRNA

To knock-down BC1 lncRNA, four different siRNAs corresponding to nucleotides 98–116 (siBC1–1: 5′-AAGACAAAAUAACAAAAAG-3′), 110–128 (siBC1–2: 5′-CAAAAAGACCAAAAAAAAA-3′), 127–144 (siBC1–3: 5′-AACAAGGUAACUGGCACAC-3′) and 136–154 (siBC1–4: 5′-ACUGGCACACACAACCUUU-3′) were designed. Scrambled sequence (siNC: 5′-CCUCGUGCCGUUCCAUCAGGUAG-3′ was used as a control.

### Quantitative real time PCR (qRT-PCR) assay

Total RNA (2 μg) was reverse-transcribed using a reverse transcriptase (Invitrogen, Carlsbad, CA) for quantitative reverse transcription PCR (qRT-PCR) analysis. The PCRs were performed in triplicate using an ABI^®^ 7500 (Life Technologies, Carlsbad, CA) with SYBR RT-PCR kit (Takara Bio Inc., Shiga, Japan). The reactions were incubated at 95 °C for 1 min, and 40 cycles of 95 °C for 5 sec and 60 °C for 34 sec. PCR primers for BC1 were 5′-TGGGGATTTAGCTCAGTGGTAG-3′ (forward) and 5′-GTTGTGTGTGCCAGTTACCTTG-3′ (reverse). Data were normalized to β-actin expression.

### Synthesis of PNA and DNA oligomer

The PNA probe was synthesized by Panagene, Inc. (Daejeon, Korea) and the DNA oligomer were purchased from Bioneer (Daejeon, Korea). All PNA probes were 22-mer in length and were labeled with FAM fluorescent dye. The sequences of the DNA oligomer were 5′-AAGACAAAATAACAAAAAGACC-3′ (FAM-PNA-BC1–1 complementary oligomer) and 5′-AACAAGGTAACTGGCACACACA-3′ (FAM-PNA-BC1–3 complementary oligomer).

### Preparation of GO

The GO was exfoliated onto the form of GO-COO^−^ sheet by ultrasonification on ice chamber. The brown dispersion was centrifuged at 3,470 g for 5 min to discard unexfoliated powder. GO-NH_3_^+^ was prepared via the amine exchange reaction using 1–[3-(dimethylamino) propyl]-3-ethylcarbodiimide methyliodide and ethylene diamine (EDC). After EDC was treated into the prepared GO-COO^−^, the reactive mixture was stirred for 12 h, and dialyzed for 4 days in a membrane tube to remove residual chemicals.

### Specificity of FAM-PNA-BC1-GO complex

Each fluorescent PNA probe (40 pmol per probe) was mixed with the GO in a 100 μL of buffer (Tris-HCl, pH 7.5) for 10 min at room temperature. The quenched fluorescence signals were monitored after the formation of PNA probe-GO complex. A total of FAM-PNA-GO complex solution was mixed with target oligomer having a complementary sequence, followed by addition of various concentrations of oligomer. Fluorescence measurements were carried out using a Varioskan Flash Multimode Reader (Thermo Fisher Scientific, Inc., Waltham, MA). The fluorescent plate images were obtained using an IVIS^®^ Lumina II (Perkin Elmer, Waltham, MA).

### WST-8 viability assay

Cell viability was measured using a cell counting kit-8 (CCK-8, Dojindo, Rockville, MD). Cells were seeded on 48-well cell culture plate at 2 × 10^4^ cells per well and maintained for 24 h. The cells were washed with opti-MEM and were incubated with GO (concentration range of 0–200 μg/mL) in opti-MEM. After 14 h exposure, the cells were rinsed with opti-MEM and 20 μL of CCK-8 solution was added to each well containing 200 μL of opti-MEM. One hour after incubation, 100 μL of the mixture was transferred to another 96-well plate and the absorbance of the mixture solutions was measured at 450 nm using a GloMax^®^-Multi Detection System (Promega, Fitchburg, WI).

### BC1 lncRNA detection in cells

Cells were seeded on 24-well cell culture plate at 4 × 10^4^ cells per well and maintained for 24 h. Each fluorescent PNA probe (100 pmol per probe) was mixed with the GO (1 μg) in a 250 μL of opti-MEM media (Invitrogen, Carlsbad, CA) for 10 min at room temperature. The media were discarded and FAM-PNA-BC1-GO complexes were treated to cells for 14 h at 37 °C. After the cells were fixed with 3.7% paraformaldehyde solution, confocal images were obtained using a LSM510 confocal laser scanning microscope (Carl Zeiss Inc., Jena, Germany).

## Additional Information

**How to cite this article**: Kim, M. Y. *et al.* Detection of intra-brain cytoplasmic 1 (BC1) long noncoding RNA using graphene oxide-fluorescence beacon detector. *Sci. Rep.* **6**, 22552; doi: 10.1038/srep22552 (2016).

## Supplementary Material

Supplementary Information

## Figures and Tables

**Figure 1:**
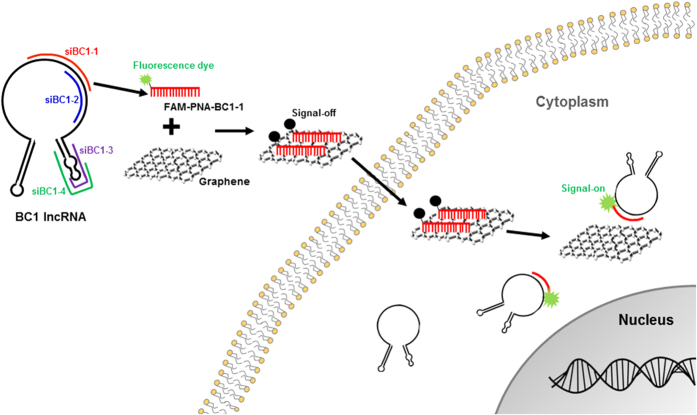
Schematic representation of GO-based sensor for the detection of BC1 lncRNA. Fluorescent dye (FAM)-labeled PNA probe based on sequence of siRNA was loaded with GO and quenched FAM-PNA-GO was internalized into neural stem cells. The fluorescence signal was recovered after fluorescent dye-labeled PNA probe was released from GO by interaction with complementary target BC1 lncRNA.
