# Supplementary material for: Detection of intra-brain cytoplasmic 1 (BC1) long noncoding RNA using graphene oxide-fluorescence beacon detector
Source: Sci Rep. 2016 Mar 21;6:22552. doi: 10.1038/srep22552 (PMC4800405; doi:10.1038/srep22552)
Supplement: Supplementary Information [file srep22552-s1.pdf]

Supplementary Information

Detection of intra-brain cytoplasmic 1 (BC1) long noncoding RNA using graphene oxide-fluorescence beacon detector

Mee Young Kim[1,+], Do Won Hwang[1,2,+,*], Fangyuan Li[3,+], Yoori Choi[1], Jung Woo Byun[1], Dongho Kim[4], Jee-Eun Kim[4], Kookheon Char[3,*] & Dong Soo Lee[1,2,*]

Supplementary Table. Sequence information of peptide nucleic acid (PNA) probe for BC1 lncRNA.

Probe	Sequence	Length	GC (%)
FAM-PNA-scr 1	FAM-OO-TTCTGTTTTATTGTTTTTCTGG	22	27.27
FAM-PNA-BC1-1	FAM-OO-GGTCTTTTTGTTATTTTGTCTT	22	27.27
FAM-PNA-BC1-3	FAM-OO-TGTGTGTGCCAGTTACCTTGTT	22	45.45

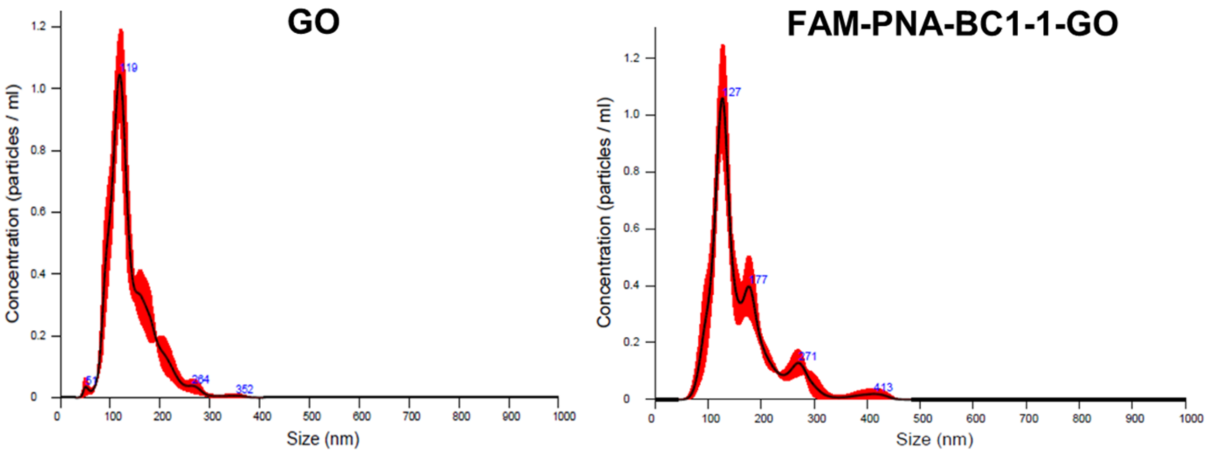

Supplementary Figure S1. Nanoparticle Tracking Analysis was used to determine the size of GO and FAM-PNA-BC1-1-GO. Size distribution and maximum peak was similar between two groups.

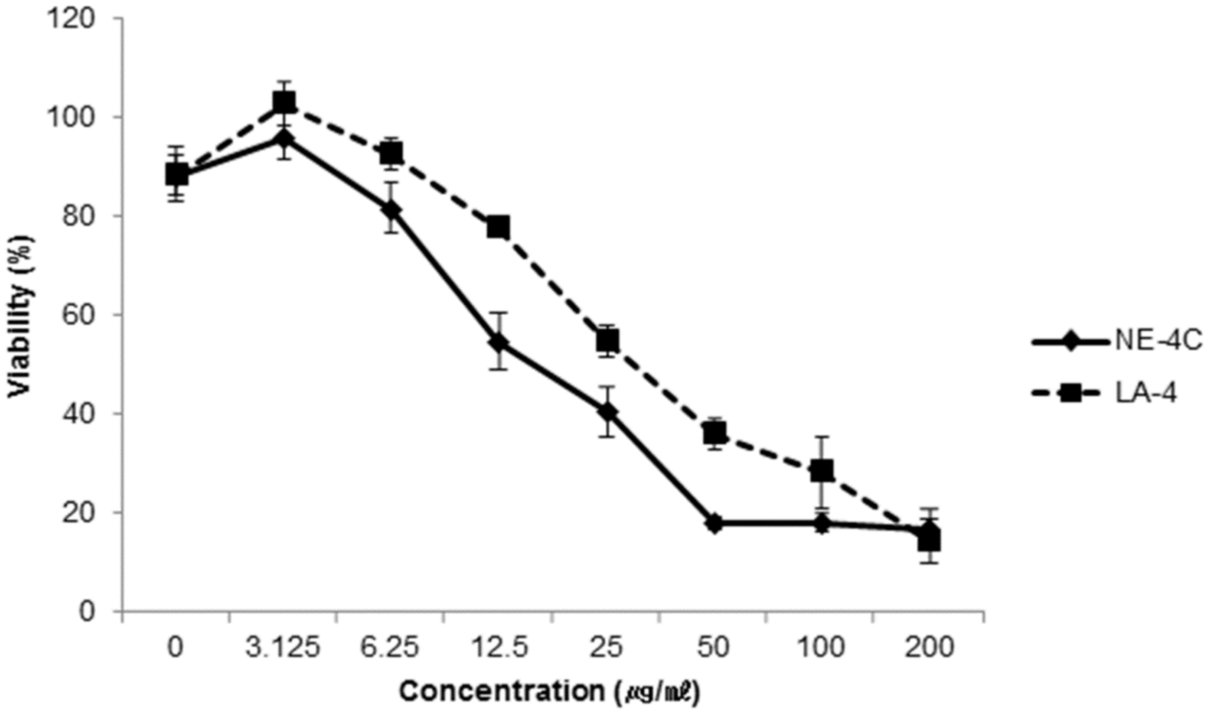

Supplementary Figure S2. Cytotoxicity of GO in the cell lines. The viabilities of cells incubated with different concentrations of GO for 14 h were measured by using WST-8 assay. Each data represents the mean values from five independent experiments.

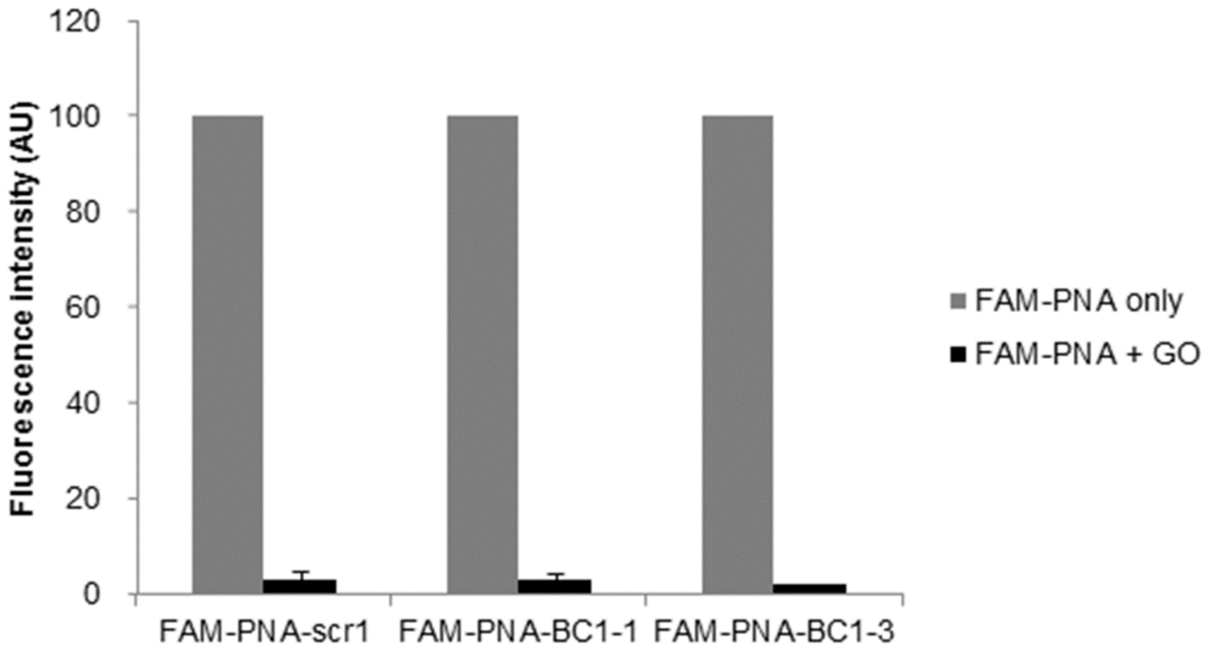

Supplementary Figure S3. Quenched fluorescence intensity of FAM-PNA probes in opti-MEM. FAM-conjugated PNA probes were reacted with or without GO in opti-MEM buffer. Fluorescence signals of FAM-PNA probes were quenched after incubation with GO within 10 minutes, measured by fluorometer.

A

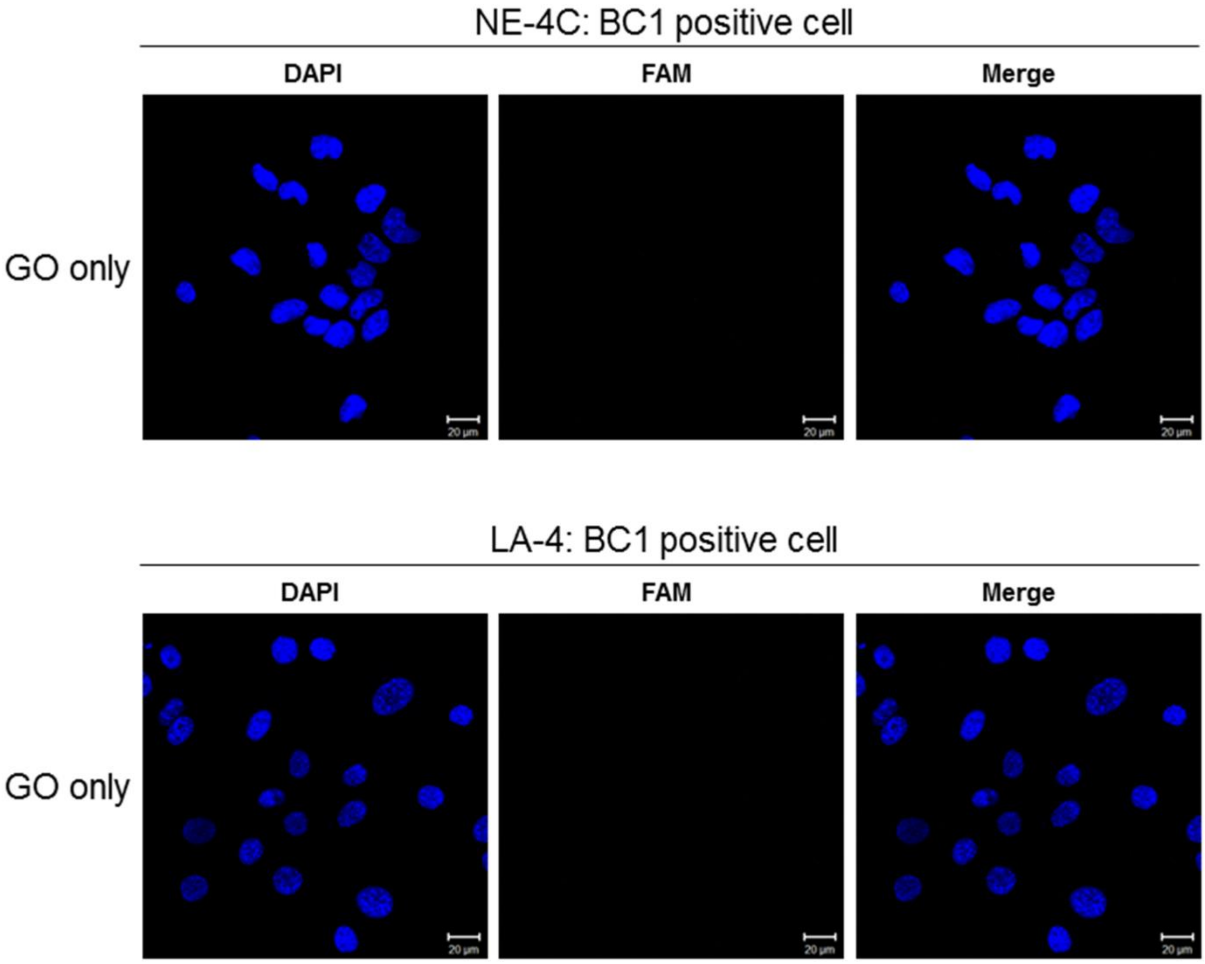
NE-4C: BC1 positive cell
DAPI
FAM
Merge
GO only
20 μm
20 μm
20 μm
LA-4: BC1 positive cell
DAPI
FAM
Merge
GO only
20 μm
20 μm
20 μm

B

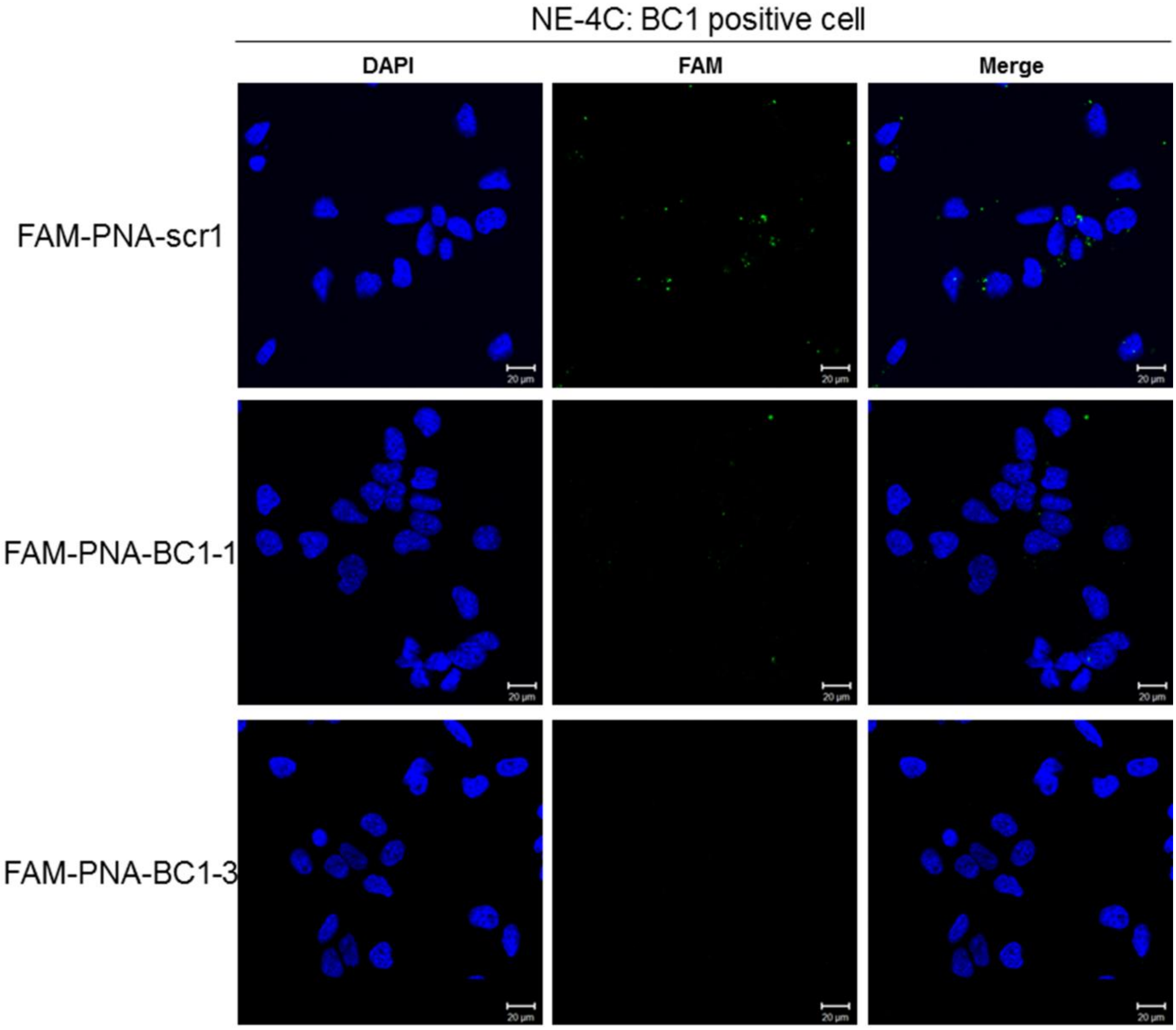

NE-4C: BC1 positive cell
DAPI
FAM
Merge
FAM-PNA-scr1
FAM-PNA-BC1-1
FAM-PNA-BC1-3
20 μm

C

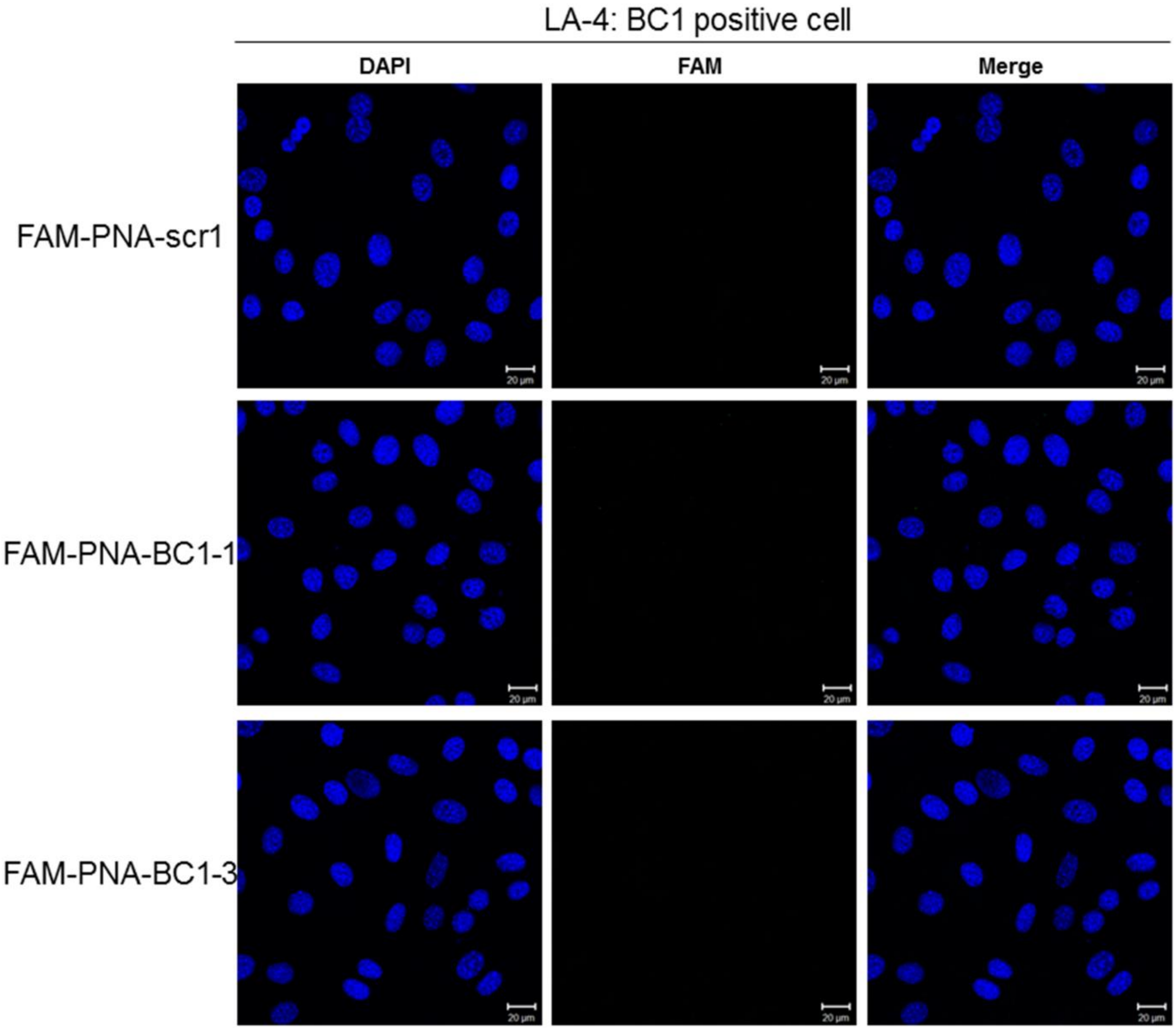

Supplementary Figure S4. The fluorescent images of the cells treated with FAM-conjugated PNA probes alone or GO alone. (**A**) No fluorescence signals were observed after the cells were incubated with GO (0.4 μg) alone for 14 h. (**B, C**) Fluorescent images of the cells were taken 14 h after treatment of FAM-conjugated PNA probes (100 pmol) alone in NE-4C (**B**) and LA-4 (**C**). Scale bar: 20 μm. (Green, FAM signals from PNA probe; blue, DAPI signal from nuclei).
